# 2,4-Bis[(3-allyl­imidazolium-1-yl)meth­yl]mesitylene bis­(hexa­fluoridophosphate)

**DOI:** 10.1107/S1600536811027541

**Published:** 2011-07-16

**Authors:** Rosenani A. Haque, Mohammed Z. Ghdhayeb, Madhukar Hemamalini, Hoong-Kun Fun

**Affiliations:** a School of Chemical Sciences, Universiti Sains Malaysia, 11800 USM, Penang, Malaysia; b X-ray Crystallography Unit, School of Physics, Universiti Sains Malaysia, 11800 USM, Penang, Malaysia

## Abstract

In the title mol­ecular salt, C_23_H_30_N_4_^2+^·2PF_6_^−^, the central benzene ring of the cation makes dihedral angles of 89.80 (8) and 85.23 (7)° with the pendant imidazole rings. In the crystal, the cations and anions are linked by numerous C—H⋯F hydrogen bonds, thereby forming a three-dimensional network.

## Related literature

For further details of imidazol-2-ylidenes, see: Arduengo *et al.* (1991 ▶); Scott & Nolan (2005 ▶); Scholl *et al.* (1999 ▶). For a related structure, see: Villegas *et al.* (2005 ▶). For the stability of the temperature controller used in the data collection, see: Cosier & Glazer (1986 ▶).
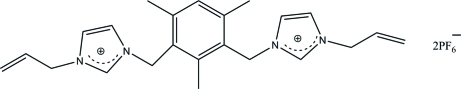


## Experimental

### 

#### Crystal data

C_23_H_30_N_4_^2+^·2PF_6_^−^
*M* *_r_* = 652.45
Monoclinic, 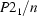

*a* = 11.9269 (4) Å
*b* = 19.1480 (6) Å
*c* = 12.4233 (4) Å
β = 103.479 (1)°
*V* = 2759.04 (15) Å^3^
*Z* = 4
Mo *K*α radiation
μ = 0.26 mm^−1^
*T* = 100 K
0.67 × 0.29 × 0.15 mm

#### Data collection

Bruker SMART APEXII CCD diffractometer
Absorption correction: multi-scan (*SADABS*; Bruker, 2009 ▶) *T*_min_ = 0.845, *T*_max_ = 0.961
67401 measured reflections
9961 independent reflections
8004 reflections with *I* > 2σ(*I*)
*R*_int_ = 0.030

#### Refinement

*R*[*F* ^2^ > 2σ(*F* ^2^)] = 0.046
*wR*(*F* ^2^) = 0.117
*S* = 1.05
9961 reflections
397 parameters
H atoms treated by a mixture of independent and constrained refinement
Δρ_max_ = 0.94 e Å^−3^
Δρ_min_ = −0.38 e Å^−3^

### 

Data collection: *APEX2* (Bruker, 2009 ▶); cell refinement: *SAINT* (Bruker, 2009 ▶); data reduction: *SAINT*; program(s) used to solve structure: *SHELXTL* (Sheldrick, 2008 ▶); program(s) used to refine structure: *SHELXTL*; molecular graphics: *SHELXTL*; software used to prepare material for publication: *SHELXTL* and *PLATON* (Spek, 2009 ▶).

## Supplementary Material

Crystal structure: contains datablock(s) global, I. DOI: 10.1107/S1600536811027541/hb5934sup1.cif

Structure factors: contains datablock(s) I. DOI: 10.1107/S1600536811027541/hb5934Isup2.hkl

Supplementary material file. DOI: 10.1107/S1600536811027541/hb5934Isup3.cml

Additional supplementary materials:  crystallographic information; 3D view; checkCIF report

## Figures and Tables

**Table 1:** Hydrogen-bond geometry (Å, °)

*D*—H⋯*A*	*D*—H	H⋯*A*	*D*⋯*A*	*D*—H⋯*A*
C1—H1*A*⋯F3^i^	1.00 (2)	2.49 (2)	3.411 (2)	153.1 (18)
C1—H2*B*⋯F7^ii^	1.01 (2)	2.47 (2)	3.480 (2)	173.7 (18)
C3—H3*A*⋯F6^ii^	0.97	2.53	3.3303 (17)	140
C3—H3*B*⋯F2^i^	0.97	2.48	3.4151 (17)	161
C4—H4*A*⋯F8^iii^	0.93	2.37	3.248 (2)	157
C5—H5*A*⋯F4^iv^	0.93	2.34	3.0754 (16)	136
C5—H5*A*⋯F12^iii^	0.93	2.52	3.1110 (18)	122
C6—H6*A*⋯F6^ii^	0.93	2.31	3.1005 (16)	143
C14—H14*A*⋯F9^iv^	0.97	2.45	3.401 (2)	167
C15—H15*A*⋯F6^ii^	0.93	2.42	3.1873 (16)	139
C16—H16*A*⋯F8^iv^	0.93	2.46	3.3113 (19)	152
C17—H17*A*⋯F3	0.93	2.53	3.2000 (18)	129
C18—H18*B*⋯F4^ii^	0.97	2.54	3.2398 (17)	129
C18—H18*B*⋯F6^ii^	0.97	2.50	3.3781 (17)	150

Symmetry codes: (i) 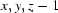; (ii) 
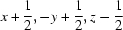
; (iii) 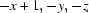; (iv) 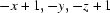.

**Fig. 1.**
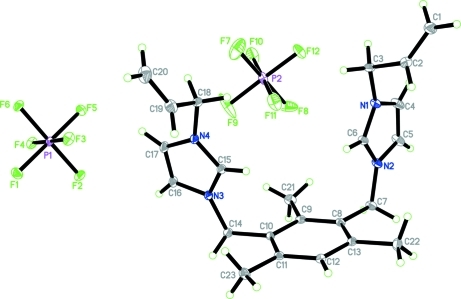
The asymmetric unit of the title compound, showing 30% probability displacement ellipsoids.

**Fig. 2.**
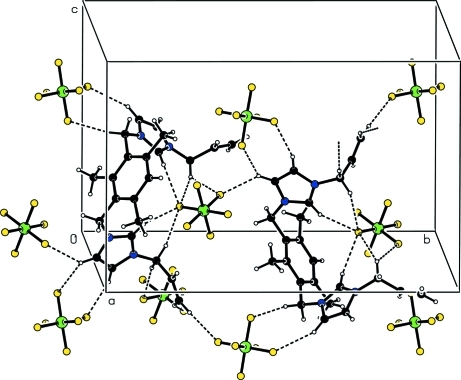
The packing of the title compound, showing the hydrogen-bonded (dashed lines) network.

## supplementary crystallographic information

### Comment

Since Arduengo's report of stable imidazol-2-ylidenes (Arduengo *et al.*, 1991), there has been growing interest in the use of *N*-heterocyclic carbene (NHC) species (Scott & Nolan, 2005). NHC ligands act as σ-donor ligands with minimal π-accepting. NHC ligands have proved to be particularly useful in olefin metathesis and palladium-catalyzed cross-coupling reactions. Imidazol-2-ylidene and imidazolin-2-ylidene-based ruthenium alkylidenes are more active and thermally stable than the original tricyclohexylphosphine-based systems developed by Scholl *et al.*, (1999).  The title compound (I), which possesses an imidazolidine ring, is a member of this NHC family.

The asymmetric unit of the title compound, (Fig. 1), consists of one 2,4-Bis(3-allylimidazolium-1-ylmethyl)mesityleninium dication and two hexafluorophosphate anions.  The central benzene (C8–C13) ring makes dihedral angles of 89.80 (8)° and 85.23 (7)° with the terminal imidazole (N1/N2/C4–C6)/(N3/N4/C15–C17) rings.  The P–F distances in the anion are in the range 1.5906 (9)–1.6161 (9) Å.  This values agree with a previously reported crystal structure (Villegas *et al.*, 2005).

In the crystal (Fig. 2) of (I), the cations and anions are linked *via* intermolecular C—H···F (Table 1) hydrogen bonds forming a three-dimensional network.

### Experimental

A mixture of imidazole (0.9 g, 13.2 mmol) and sodium hydroxide (0.5 g, 12 mmol) in DMSO (5 ml) was heated to 90°C for 2 hr. The mixture was cooled to room temperature using a water bath. To this mixture, a solution of 2,4-bis(bromomethyl) mesitylene (2 g, 6.5 mmol) in DMSO (10 ml) was added. The mixture was then heated to 40°C for 1 hr, then poured into water (40 ml) followed by cooling in ice. The precipitate formed was collected, washed with water, and recrystallized from methanol/water to give product A (1,3-bis(*N*-imidazole-1-yl methyl) benzene) as a white solid (1.39 g, 56%). Furthermore, a mixture of A (0.5 g, 1.3 mmol) and allyl bromide (0.4 g, 3.3 mmol) in acetonitrile (30 ml) was refluxed at 90°C for 24 hr. The solvent was removed under reduced pressure to give a pale-brown oil.  The resulted bromide salt was converted to its hexafluorophosphate salt by metathesis reaction using KPF_6_ (0.2g, 1.1 mmol) in 20 ml of methanol. The precipitate formed was collected and washed with distilled water (2 × 5 ml) and then recrystallized from acetonitrile to give colorless solid (0.41g, 87%).  Colourless blocks of (I) were obtained by slow evaporation of the salt solution in acetonitrile at room temperature.

### Refinement

Atoms H1A, H2A, H2B, H19A, H20A and H20B were located from a difference Fourier maps and refined freely [C–H = 0.96 (2)–1.01 (2) Å]. The remaining H atoms were positioned geometrically [C–H = 0.93–0.97 Å] and were refined using a riding model, with *U*_iso_(H) = 1.2 or 1.5 *U*_eq_(C). A rotating group model was used for the methyl group. The highest residual electron density peak is located at 0.78 Å from P1 and the deepest hole 0.56 Å located at from P2.

### Crystal data

**Table:**

C_23_H_30_N_4_^2+^·2PF_6_^−^	*F*(000) = 1336
*M**_r_* = 652.45	*D*_x_ = 1.571 Mg m^−^^3^
Monoclinic, *P*2_1_/*n*	Mo *K*α radiation, λ = 0.71073 Å
Hall symbol: -P 2yn	Cell parameters from 9948 reflections
*a* = 11.9269 (4) Å	θ = 2.7–32.5°
*b* = 19.1480 (6) Å	µ = 0.26 mm^−^^1^
*c* = 12.4233 (4) Å	*T* = 100 K
β = 103.479 (1)°	Block, colourless
*V* = 2759.04 (15) Å^3^	0.67 × 0.29 × 0.15 mm
*Z* = 4	

### Data collection

**Table:**

Bruker SMART APEXII CCD diffractometer	9961 independent reflections
Radiation source: fine-focus sealed tube	8004 reflections with *I* > 2σ(*I*)
graphite	*R*_int_ = 0.030
φ and ω scans	θ_max_ = 32.7°, θ_min_ = 2.0°
Absorption correction: multi-scan (*SADABS*; Bruker, 2009)	*h* = −17→11
*T*_min_ = 0.845, *T*_max_ = 0.961	*k* = −29→28
67401 measured reflections	*l* = −18→18

### Refinement

**Table:**

Refinement on *F*^2^	Primary atom site location: structure-invariant direct methods
Least-squares matrix: full	Secondary atom site location: difference Fourier map
*R*[*F*^2^ > 2σ(*F*^2^)] = 0.046	Hydrogen site location: inferred from neighbouring sites
*wR*(*F*^2^) = 0.117	H atoms treated by a mixture of independent and constrained refinement
*S* = 1.05	*w* = 1/[σ^2^(*F*_o_^2^) + (0.0492*P*)^2^ + 1.8158*P*] where *P* = (*F*_o_^2^ + 2*F*_c_^2^)/3
9961 reflections	(Δ/σ)_max_ < 0.001
397 parameters	Δρ_max_ = 0.94 e Å^−^^3^
0 restraints	Δρ_min_ = −0.38 e Å^−^^3^

### Special details

**Table:**

Experimental. The crystal was placed in the cold stream of an Oxford Cryosystems Cobra open-flow nitrogen cryostat (Cosier & Glazer, 1986) operating at 100.0 (1) K.
Geometry. All s.u.'s (except the s.u. in the dihedral angle between two l.s. planes) are estimated using the full covariance matrix. The cell s.u.'s are taken into account individually in the estimation of s.u.'s in distances, angles and torsion angles; correlations between s.u.'s in cell parameters are only used when they are defined by crystal symmetry. An approximate (isotropic) treatment of cell s.u.'s is used for estimating s.u.'s involving l.s. planes.
Refinement. Refinement of F^2^ against ALL reflections. The weighted R-factor wR and goodness of fit S are based on F^2^, conventional R-factors R are based on F, with F set to zero for negative F^2^. The threshold expression of F^2^ > 2σ(F^2^) is used only for calculating R-factors(gt) etc. and is not relevant to the choice of reflections for refinement. R-factors based on F^2^ are statistically about twice as large as those based on F, and R- factors based on ALL data will be even larger.

### Fractional atomic coordinates and isotropic or equivalent isotropic displacement parameters (Å^2^)

**Table:**

	*x*	*y*	*z*	*U*_iso_*/*U*_eq_	
P1	0.29845 (3)	0.210329 (17)	0.80189 (3)	0.01483 (7)	
F1	0.35585 (8)	0.23350 (5)	0.92540 (7)	0.02644 (19)	
F2	0.39126 (7)	0.14866 (5)	0.80923 (8)	0.02345 (18)	
F3	0.38042 (8)	0.26307 (5)	0.75519 (8)	0.02462 (18)	
F4	0.21375 (8)	0.15861 (4)	0.84670 (8)	0.02257 (18)	
F5	0.23971 (8)	0.18722 (5)	0.67721 (7)	0.02409 (18)	
F6	0.20405 (7)	0.27188 (4)	0.79326 (7)	0.02129 (17)	
P2	0.39326 (4)	0.09732 (2)	0.23406 (3)	0.02263 (9)	
F7	0.33168 (13)	0.17019 (7)	0.23935 (15)	0.0666 (4)	
F8	0.45444 (12)	0.02332 (6)	0.22843 (11)	0.0535 (4)	
F9	0.39508 (16)	0.08293 (9)	0.36090 (9)	0.0744 (6)	
F10	0.27072 (10)	0.05948 (7)	0.19677 (10)	0.0473 (3)	
F11	0.51578 (10)	0.13420 (6)	0.27155 (9)	0.0408 (3)	
F12	0.39313 (10)	0.10992 (6)	0.10809 (8)	0.0338 (2)	
N1	0.66628 (10)	0.10107 (6)	0.07606 (9)	0.0179 (2)	
N2	0.77406 (11)	0.02414 (6)	0.17799 (9)	0.0183 (2)	
N3	0.64891 (10)	0.12044 (6)	0.58660 (9)	0.0156 (2)	
N4	0.54619 (10)	0.20808 (6)	0.51093 (9)	0.0159 (2)	
C1	0.66298 (15)	0.23004 (9)	−0.12513 (14)	0.0291 (3)	
C2	0.69336 (13)	0.20738 (7)	−0.02262 (13)	0.0223 (3)	
C3	0.61482 (12)	0.16890 (7)	0.03541 (12)	0.0200 (3)	
H3A	0.6026	0.1965	0.0971	0.024*	
H3B	0.5406	0.1614	−0.0153	0.024*	
C4	0.65806 (15)	0.04084 (8)	0.01475 (12)	0.0260 (3)	
H4A	0.6143	0.0345	−0.0570	0.031*	
C5	0.72544 (15)	−0.00746 (7)	0.07818 (12)	0.0263 (3)	
H5A	0.7369	−0.0533	0.0583	0.032*	
C6	0.73677 (12)	0.08990 (7)	0.17422 (11)	0.0180 (2)	
H6A	0.7568	0.1225	0.2309	0.022*	
C7	0.85691 (13)	−0.00899 (7)	0.27138 (11)	0.0211 (3)	
H7A	0.9292	−0.0171	0.2502	0.025*	
H7B	0.8269	−0.0539	0.2872	0.025*	
C8	0.87906 (12)	0.03551 (6)	0.37446 (11)	0.0159 (2)	
C9	0.80327 (11)	0.03173 (7)	0.44525 (11)	0.0162 (2)	
C10	0.82516 (11)	0.07270 (6)	0.54174 (10)	0.0146 (2)	
C11	0.92042 (11)	0.11794 (7)	0.56595 (10)	0.0153 (2)	
C12	0.99366 (11)	0.12044 (7)	0.49427 (11)	0.0162 (2)	
H12A	1.0575	0.1498	0.5108	0.019*	
C13	0.97463 (11)	0.08027 (7)	0.39822 (11)	0.0159 (2)	
C14	0.74462 (12)	0.06960 (7)	0.61879 (11)	0.0187 (2)	
H14A	0.7131	0.0228	0.6178	0.022*	
H14B	0.7874	0.0795	0.6937	0.022*	
C15	0.64268 (11)	0.17246 (7)	0.51443 (11)	0.0172 (2)	
H15A	0.6967	0.1823	0.4733	0.021*	
C16	0.55241 (12)	0.12253 (8)	0.63004 (12)	0.0204 (3)	
H16A	0.5349	0.0920	0.6820	0.024*	
C17	0.48838 (13)	0.17740 (8)	0.58249 (12)	0.0215 (3)	
H17A	0.4183	0.1918	0.5956	0.026*	
C18	0.51008 (12)	0.27030 (7)	0.44200 (11)	0.0189 (2)	
H18A	0.4272	0.2691	0.4133	0.023*	
H18B	0.5460	0.2696	0.3795	0.023*	
C19	0.54258 (16)	0.33656 (8)	0.50622 (13)	0.0271 (3)	
C20	0.4720 (2)	0.39046 (9)	0.50092 (17)	0.0386 (4)	
C21	0.69872 (13)	−0.01543 (8)	0.41868 (13)	0.0241 (3)	
H21A	0.6364	0.0062	0.4431	0.036*	
H21B	0.7172	−0.0593	0.4559	0.036*	
H21C	0.6763	−0.0231	0.3402	0.036*	
C22	1.05571 (13)	0.08804 (8)	0.32222 (13)	0.0239 (3)	
H22A	1.1198	0.1168	0.3571	0.036*	
H22B	1.0157	0.1094	0.2542	0.036*	
H22C	1.0833	0.0428	0.3070	0.036*	
C23	0.94331 (13)	0.16523 (8)	0.66612 (12)	0.0227 (3)	
H23A	1.0110	0.1927	0.6676	0.034*	
H23B	0.9549	0.1374	0.7321	0.034*	
H23C	0.8785	0.1956	0.6623	0.034*	
H1A	0.582 (2)	0.2231 (12)	−0.1680 (19)	0.045 (6)*	
H2A	0.7719 (18)	0.2165 (11)	0.0220 (17)	0.031 (5)*	
H2B	0.7170 (19)	0.2563 (12)	−0.1625 (18)	0.039 (6)*	
H19A	0.6209 (19)	0.3395 (11)	0.5498 (18)	0.035 (5)*	
H20A	0.498 (2)	0.4327 (13)	0.541 (2)	0.050 (7)*	
H20B	0.391 (2)	0.3861 (12)	0.456 (2)	0.045 (7)*	

### Atomic displacement parameters (Å^2^)

**Table:**

	*U*^11^	*U*^22^	*U*^33^	*U*^12^	*U*^13^	*U*^23^
P1	0.01606 (15)	0.01226 (13)	0.01678 (15)	0.00022 (11)	0.00507 (12)	−0.00065 (11)
F1	0.0319 (5)	0.0247 (4)	0.0199 (4)	−0.0002 (4)	0.0005 (4)	−0.0046 (3)
F2	0.0204 (4)	0.0201 (4)	0.0300 (4)	0.0057 (3)	0.0060 (3)	−0.0014 (3)
F3	0.0232 (4)	0.0206 (4)	0.0332 (5)	−0.0039 (3)	0.0129 (4)	0.0017 (3)
F4	0.0245 (4)	0.0154 (4)	0.0311 (4)	−0.0004 (3)	0.0132 (4)	0.0038 (3)
F5	0.0256 (4)	0.0267 (4)	0.0188 (4)	0.0009 (4)	0.0028 (3)	−0.0038 (3)
F6	0.0235 (4)	0.0159 (4)	0.0268 (4)	0.0062 (3)	0.0106 (3)	0.0037 (3)
P2	0.0322 (2)	0.01989 (16)	0.01803 (16)	−0.00999 (15)	0.01042 (15)	−0.00370 (13)
F7	0.0671 (9)	0.0405 (7)	0.0987 (12)	0.0078 (6)	0.0322 (9)	−0.0301 (7)
F8	0.0680 (8)	0.0262 (5)	0.0476 (7)	0.0109 (5)	−0.0242 (6)	−0.0042 (5)
F9	0.1209 (13)	0.0869 (11)	0.0207 (5)	−0.0730 (10)	0.0274 (7)	−0.0135 (6)
F10	0.0404 (6)	0.0615 (8)	0.0444 (7)	−0.0288 (6)	0.0185 (5)	−0.0124 (6)
F11	0.0414 (6)	0.0502 (7)	0.0307 (5)	−0.0255 (5)	0.0080 (5)	−0.0122 (5)
F12	0.0448 (6)	0.0379 (5)	0.0193 (4)	−0.0120 (5)	0.0084 (4)	0.0035 (4)
N1	0.0218 (5)	0.0155 (5)	0.0146 (5)	−0.0008 (4)	0.0008 (4)	0.0006 (4)
N2	0.0270 (6)	0.0130 (5)	0.0135 (5)	0.0009 (4)	0.0019 (4)	−0.0005 (4)
N3	0.0157 (5)	0.0170 (5)	0.0149 (5)	0.0006 (4)	0.0055 (4)	0.0021 (4)
N4	0.0164 (5)	0.0166 (5)	0.0158 (5)	0.0015 (4)	0.0060 (4)	0.0012 (4)
C1	0.0252 (7)	0.0332 (8)	0.0287 (8)	0.0002 (6)	0.0062 (6)	0.0084 (6)
C2	0.0200 (6)	0.0196 (6)	0.0263 (7)	−0.0004 (5)	0.0034 (6)	0.0014 (5)
C3	0.0201 (6)	0.0198 (6)	0.0190 (6)	0.0035 (5)	0.0020 (5)	0.0033 (5)
C4	0.0387 (8)	0.0184 (6)	0.0161 (6)	−0.0054 (6)	−0.0029 (6)	−0.0022 (5)
C5	0.0446 (9)	0.0143 (6)	0.0165 (6)	−0.0024 (6)	−0.0002 (6)	−0.0036 (5)
C6	0.0233 (6)	0.0149 (5)	0.0142 (5)	0.0022 (5)	0.0014 (5)	−0.0015 (4)
C7	0.0290 (7)	0.0158 (6)	0.0162 (6)	0.0064 (5)	0.0003 (5)	−0.0014 (5)
C8	0.0203 (6)	0.0120 (5)	0.0139 (5)	0.0040 (4)	0.0009 (5)	0.0002 (4)
C9	0.0167 (5)	0.0126 (5)	0.0176 (6)	0.0007 (4)	0.0006 (5)	0.0015 (4)
C10	0.0154 (5)	0.0140 (5)	0.0143 (5)	0.0023 (4)	0.0035 (4)	0.0023 (4)
C11	0.0168 (5)	0.0134 (5)	0.0145 (5)	0.0024 (4)	0.0014 (5)	0.0004 (4)
C12	0.0148 (5)	0.0155 (5)	0.0176 (6)	0.0004 (4)	0.0022 (5)	0.0007 (4)
C13	0.0167 (5)	0.0148 (5)	0.0165 (5)	0.0041 (4)	0.0045 (5)	0.0033 (4)
C14	0.0192 (6)	0.0195 (6)	0.0183 (6)	0.0042 (5)	0.0063 (5)	0.0060 (5)
C15	0.0167 (6)	0.0186 (6)	0.0176 (6)	0.0019 (5)	0.0070 (5)	0.0039 (5)
C16	0.0206 (6)	0.0228 (6)	0.0210 (6)	0.0006 (5)	0.0116 (5)	0.0039 (5)
C17	0.0211 (6)	0.0249 (7)	0.0221 (6)	0.0029 (5)	0.0122 (5)	0.0030 (5)
C18	0.0197 (6)	0.0188 (6)	0.0190 (6)	0.0044 (5)	0.0060 (5)	0.0033 (5)
C19	0.0379 (9)	0.0208 (6)	0.0235 (7)	−0.0014 (6)	0.0090 (7)	0.0013 (5)
C20	0.0603 (13)	0.0220 (7)	0.0392 (10)	0.0048 (8)	0.0230 (10)	0.0002 (7)
C21	0.0229 (7)	0.0219 (6)	0.0254 (7)	−0.0062 (5)	0.0015 (6)	−0.0007 (5)
C22	0.0254 (7)	0.0252 (7)	0.0240 (7)	0.0050 (6)	0.0117 (6)	0.0035 (5)
C23	0.0257 (7)	0.0222 (6)	0.0190 (6)	0.0000 (5)	0.0028 (5)	−0.0057 (5)

### Geometric parameters (Å, °)

**Table:**

P1—F1	1.5906 (9)	C7—H7A	0.9700
P1—F4	1.6040 (9)	C7—H7B	0.9700
P1—F3	1.6052 (9)	C8—C13	1.4015 (19)
P1—F5	1.6063 (9)	C8—C9	1.4022 (19)
P1—F2	1.6066 (9)	C9—C10	1.4053 (18)
P1—F6	1.6161 (9)	C9—C21	1.5124 (19)
P2—F12	1.5830 (10)	C10—C11	1.4044 (18)
P2—F7	1.5853 (13)	C10—C14	1.5070 (18)
P2—F11	1.5918 (11)	C11—C12	1.3860 (19)
P2—F9	1.5949 (12)	C11—C23	1.5116 (18)
P2—F10	1.6000 (11)	C12—C13	1.3931 (19)
P2—F8	1.6029 (12)	C12—H12A	0.9300
N1—C6	1.3273 (17)	C13—C22	1.5081 (19)
N1—C4	1.3729 (18)	C14—H14A	0.9700
N1—C3	1.4746 (17)	C14—H14B	0.9700
N2—C6	1.3325 (17)	C15—H15A	0.9300
N2—C5	1.3801 (18)	C16—C17	1.351 (2)
N2—C7	1.4802 (17)	C16—H16A	0.9300
N3—C15	1.3306 (17)	C17—H17A	0.9300
N3—C16	1.3810 (17)	C18—C19	1.501 (2)
N3—C14	1.4825 (17)	C18—H18A	0.9700
N4—C15	1.3297 (17)	C18—H18B	0.9700
N4—C17	1.3771 (17)	C19—C20	1.324 (2)
N4—C18	1.4719 (17)	C19—H19A	0.97 (2)
C1—C2	1.314 (2)	C20—H20A	0.96 (3)
C1—H1A	1.00 (2)	C20—H20B	1.00 (2)
C1—H2B	1.01 (2)	C21—H21A	0.9600
C2—C3	1.502 (2)	C21—H21B	0.9600
C2—H2A	0.99 (2)	C21—H21C	0.9600
C3—H3A	0.9700	C22—H22A	0.9600
C3—H3B	0.9700	C22—H22B	0.9600
C4—C5	1.352 (2)	C22—H22C	0.9600
C4—H4A	0.9300	C23—H23A	0.9600
C5—H5A	0.9300	C23—H23B	0.9600
C6—H6A	0.9300	C23—H23C	0.9600
C7—C8	1.5093 (18)		
			
F1—P1—F4	90.10 (5)	N2—C7—H7B	109.2
F1—P1—F3	90.67 (5)	C8—C7—H7B	109.2
F4—P1—F3	178.56 (5)	H7A—C7—H7B	107.9
F1—P1—F5	179.61 (6)	C13—C8—C9	120.73 (12)
F4—P1—F5	89.78 (5)	C13—C8—C7	119.79 (12)
F3—P1—F5	89.44 (5)	C9—C8—C7	119.48 (12)
F1—P1—F2	90.82 (5)	C8—C9—C10	119.18 (12)
F4—P1—F2	90.45 (5)	C8—C9—C21	120.87 (12)
F3—P1—F2	90.76 (5)	C10—C9—C21	119.95 (12)
F5—P1—F2	89.55 (5)	C11—C10—C9	120.47 (12)
F1—P1—F6	89.83 (5)	C11—C10—C14	119.47 (12)
F4—P1—F6	89.31 (5)	C9—C10—C14	120.04 (12)
F3—P1—F6	89.47 (5)	C12—C11—C10	118.90 (12)
F5—P1—F6	89.79 (5)	C12—C11—C23	119.23 (12)
F2—P1—F6	179.30 (5)	C10—C11—C23	121.85 (12)
F12—P2—F7	90.75 (8)	C11—C12—C13	122.00 (12)
F12—P2—F11	90.63 (6)	C11—C12—H12A	119.0
F7—P2—F11	90.00 (8)	C13—C12—H12A	119.0
F12—P2—F9	178.63 (9)	C12—C13—C8	118.70 (12)
F7—P2—F9	90.62 (10)	C12—C13—C22	118.79 (12)
F11—P2—F9	89.28 (7)	C8—C13—C22	122.49 (12)
F12—P2—F10	89.57 (6)	N3—C14—C10	111.56 (10)
F7—P2—F10	90.56 (8)	N3—C14—H14A	109.3
F11—P2—F10	179.40 (8)	C10—C14—H14A	109.3
F9—P2—F10	90.51 (7)	N3—C14—H14B	109.3
F12—P2—F8	89.27 (7)	C10—C14—H14B	109.3
F7—P2—F8	179.50 (8)	H14A—C14—H14B	108.0
F11—P2—F8	90.50 (7)	N4—C15—N3	108.54 (11)
F9—P2—F8	89.36 (9)	N4—C15—H15A	125.7
F10—P2—F8	88.94 (7)	N3—C15—H15A	125.7
C6—N1—C4	108.81 (12)	C17—C16—N3	106.82 (12)
C6—N1—C3	125.49 (12)	C17—C16—H16A	126.6
C4—N1—C3	125.43 (12)	N3—C16—H16A	126.6
C6—N2—C5	108.41 (12)	C16—C17—N4	107.27 (12)
C6—N2—C7	126.08 (11)	C16—C17—H17A	126.4
C5—N2—C7	125.47 (11)	N4—C17—H17A	126.4
C15—N3—C16	108.72 (11)	N4—C18—C19	111.76 (12)
C15—N3—C14	126.29 (11)	N4—C18—H18A	109.3
C16—N3—C14	124.97 (11)	C19—C18—H18A	109.3
C15—N4—C17	108.65 (11)	N4—C18—H18B	109.3
C15—N4—C18	124.60 (11)	C19—C18—H18B	109.3
C17—N4—C18	126.74 (11)	H18A—C18—H18B	107.9
C2—C1—H1A	119.1 (14)	C20—C19—C18	123.37 (17)
C2—C1—H2B	123.2 (13)	C20—C19—H19A	120.2 (13)
H1A—C1—H2B	117.6 (18)	C18—C19—H19A	116.3 (13)
C1—C2—C3	124.43 (14)	C19—C20—H20A	120.1 (15)
C1—C2—H2A	119.8 (12)	C19—C20—H20B	119.0 (14)
C3—C2—H2A	115.7 (12)	H20A—C20—H20B	121 (2)
N1—C3—C2	109.89 (12)	C9—C21—H21A	109.5
N1—C3—H3A	109.7	C9—C21—H21B	109.5
C2—C3—H3A	109.7	H21A—C21—H21B	109.5
N1—C3—H3B	109.7	C9—C21—H21C	109.5
C2—C3—H3B	109.7	H21A—C21—H21C	109.5
H3A—C3—H3B	108.2	H21B—C21—H21C	109.5
C5—C4—N1	107.18 (12)	C13—C22—H22A	109.5
C5—C4—H4A	126.4	C13—C22—H22B	109.5
N1—C4—H4A	126.4	H22A—C22—H22B	109.5
C4—C5—N2	106.98 (12)	C13—C22—H22C	109.5
C4—C5—H5A	126.5	H22A—C22—H22C	109.5
N2—C5—H5A	126.5	H22B—C22—H22C	109.5
N1—C6—N2	108.62 (11)	C11—C23—H23A	109.5
N1—C6—H6A	125.7	C11—C23—H23B	109.5
N2—C6—H6A	125.7	H23A—C23—H23B	109.5
N2—C7—C8	112.08 (11)	C11—C23—H23C	109.5
N2—C7—H7A	109.2	H23A—C23—H23C	109.5
C8—C7—H7A	109.2	H23B—C23—H23C	109.5
			
C6—N1—C3—C2	89.28 (17)	C9—C10—C11—C23	177.05 (12)
C4—N1—C3—C2	−84.05 (17)	C14—C10—C11—C23	−1.50 (18)
C1—C2—C3—N1	123.60 (16)	C10—C11—C12—C13	1.00 (19)
C6—N1—C4—C5	0.02 (19)	C23—C11—C12—C13	−177.38 (12)
C3—N1—C4—C5	174.28 (14)	C11—C12—C13—C8	−0.70 (19)
N1—C4—C5—N2	0.06 (19)	C11—C12—C13—C22	177.63 (12)
C6—N2—C5—C4	−0.12 (19)	C9—C8—C13—C12	0.68 (19)
C7—N2—C5—C4	−178.04 (14)	C7—C8—C13—C12	−179.55 (11)
C4—N1—C6—N2	−0.10 (17)	C9—C8—C13—C22	−177.59 (12)
C3—N1—C6—N2	−174.35 (13)	C7—C8—C13—C22	2.18 (19)
C5—N2—C6—N1	0.14 (17)	C15—N3—C14—C10	−12.72 (19)
C7—N2—C6—N1	178.04 (13)	C16—N3—C14—C10	169.41 (12)
C6—N2—C7—C8	11.4 (2)	C11—C10—C14—N3	90.40 (14)
C5—N2—C7—C8	−171.03 (14)	C9—C10—C14—N3	−88.16 (15)
N2—C7—C8—C13	−95.56 (15)	C17—N4—C15—N3	−0.55 (16)
N2—C7—C8—C9	84.21 (15)	C18—N4—C15—N3	178.83 (12)
C13—C8—C9—C10	−0.98 (19)	C16—N3—C15—N4	0.54 (16)
C7—C8—C9—C10	179.26 (11)	C14—N3—C15—N4	−177.62 (12)
C13—C8—C9—C21	178.76 (12)	C15—N3—C16—C17	−0.32 (16)
C7—C8—C9—C21	−1.00 (18)	C14—N3—C16—C17	177.88 (13)
C8—C9—C10—C11	1.27 (18)	N3—C16—C17—N4	−0.02 (17)
C21—C9—C10—C11	−178.47 (12)	C15—N4—C17—C16	0.35 (17)
C8—C9—C10—C14	179.82 (11)	C18—N4—C17—C16	−179.02 (13)
C21—C9—C10—C14	0.07 (18)	C15—N4—C18—C19	−96.85 (16)
C9—C10—C11—C12	−1.28 (18)	C17—N4—C18—C19	82.41 (18)
C14—C10—C11—C12	−179.83 (11)	N4—C18—C19—C20	−135.43 (16)

### Hydrogen-bond geometry (Å, °)

**Table:**

*D*—H···*A*	*D*—H	H···*A*	*D*···*A*	*D*—H···*A*
C1—H1A···F3^i^	1.00 (2)	2.49 (2)	3.411 (2)	153.1 (18)
C1—H2B···F7^ii^	1.01 (2)	2.47 (2)	3.480 (2)	173.7 (18)
C3—H3A···F6^ii^	0.97	2.53	3.3303 (17)	140
C3—H3B···F2^i^	0.97	2.48	3.4151 (17)	161
C4—H4A···F8^iii^	0.93	2.37	3.248 (2)	157
C5—H5A···F4^iv^	0.93	2.34	3.0754 (16)	136
C5—H5A···F12^iii^	0.93	2.52	3.1110 (18)	122
C6—H6A···F6^ii^	0.93	2.31	3.1005 (16)	143
C14—H14A···F9^iv^	0.97	2.45	3.401 (2)	167
C15—H15A···F6^ii^	0.93	2.42	3.1873 (16)	139
C16—H16A···F8^iv^	0.93	2.46	3.3113 (19)	152
C17—H17A···F3	0.93	2.53	3.2000 (18)	129
C18—H18B···F4^ii^	0.97	2.54	3.2398 (17)	129
C18—H18B···F6^ii^	0.97	2.50	3.3781 (17)	150

Symmetry codes: (i) *x*, *y*, *z*−1; (ii) *x*+1/2, −*y*+1/2, *z*−1/2; (iii) −*x*+1, −*y*, −*z*; (iv) −*x*+1, −*y*, −*z*+1.
